# pyPPG: a Python toolbox for comprehensive photoplethysmography signal analysis

**DOI:** 10.1088/1361-6579/ad33a2

**Published:** 2024-04-08

**Authors:** Márton Á Goda, Peter H Charlton, Joachim A Behar

**Affiliations:** 1 Faculty of Biomedical Engineering, Technion Institute of Technology, Technion-IIT, Haifa, 32000, Israel; 2 Pázmány Péter Catholic University Faculty of Information Technology and Bionics, Budapest, Práter u. 50/A, 1083, Hungary; 3 Department of Public Health and Primary Care, University of Cambridge, Cambridge, CB1 8RN, United Kingdom

**Keywords:** pyPPG, photoplethysmography, beat detection, digital biomarkers

## Abstract

*Objective.* Photoplethysmography is a non-invasive optical technique that measures changes in blood volume within tissues. It is commonly and being increasingly used for a variety of research and clinical applications to assess vascular dynamics and physiological parameters. Yet, contrary to heart rate variability measures, a field which has seen the development of stable standards and advanced toolboxes and software, no such standards and limited open tools exist for continuous photoplethysmogram (PPG) analysis. Consequently, the primary objective of this research was to identify, standardize, implement and validate key digital PPG biomarkers. *Approach.* This work describes the creation of a standard Python toolbox, denoted *pyPPG*, for long-term continuous PPG time-series analysis and demonstrates the detection and computation of a high number of fiducial points and digital biomarkers using a standard fingerbased transmission pulse oximeter. *Main results.* The improved PPG peak detector had an F1-score of 88.19% for the state-of-the-art benchmark when evaluated on 2054 adult polysomnography recordings totaling over 91 million reference beats. The algorithm outperformed the open-source original Matlab implementation by ∼5% when benchmarked on a subset of 100 randomly selected MESA recordings. More than 3000 fiducial points were manually annotated by two annotators in order to validate the fiducial points detector. The detector consistently demonstrated high performance, with a mean absolute error of less than 10 ms for all fiducial points. *Significance.* Based on these fiducial points, *pyPPG* engineered a set of 74 PPG biomarkers. Studying PPG time-series variability using *pyPPG* can enhance our understanding of the manifestations and etiology of diseases. This toolbox can also be used for biomarker engineering in training data-driven models. *pyPPG* is available on https://physiozoo.com/.

## 1. Introduction

Photoplethysmography is an optical sensing technique widely used for health and fitness monitoring in clinical and consumer devices (Charlton *et al* 2022b), such as smartwatches and pulse oximeters. Photoplethysmography was developed in the 1930s (Allen 2007), and its potential value for assessing cardiovascular health was recognised in the 1940s (Dillon and Hertzman 1941). It was not until the 1970s that photoplethysmography became widely used as the sensing technology in pulse oximeters (Aoyagi 2003). Photoplethysmography-based wearable devices entered the consumer market in the 2010s (Charlton and Marozas 2022), and are now used by millions of people for unobtrusive health monitoring (Natarajan *et al* 2020).

The photoplethysmogram (PPG) signal contains a wealth of information on the heart, blood vessels, breathing and autonomic nervous system (Allen 2007). Consequently, much research is focused on extracting physiological information from the PPG (Mejia-Mejia *et al* 2022), including physiological parameters, such as blood pressure and breathing rate (Charlton *et al* 2017a, Mukkamala *et al* 2022), and disease indicators, such as vascular age and cardiovascular risk markers (Charlton *et al* 2022b). The value of photoplethysmography for heart rate and oxygen saturation monitoring is well established, its utility for detecting atrial fibrillation has recently been demonstrated (Perez *et al* 2019), and its potential to detect other diseases, such as sleep apneas and peripheral arterial disease, is being researched (Charlton *et al* 2022b).

### 1.1. The PPG signal

The PPG signal is an optical measurement of the arterial pulse wave (Charlton *et al* 2019), i.e. the wave generated when blood is ejected from the heart, temporarily increasing arterial pressure and causing vessel expansion and contraction (Alastruey *et al* 2023). Consequently, the PPG signal is influenced by a range of physiological systems, such as the heart, including heart rate, heart rhythm and the nature of ejection (Charlton *et al* 2022b), the blood vessels, including vessel stiffness, diameter and blood pressure (Charlton *et al* 2022b), the microvasculature, including peripheral compliance and resistance (Charlton *et al* 2022b), the autonomic nervous system, which influences heart rate variability (Gil *et al* 2010) and the respiratory system, which impacts the pulse wave through changes in intrathoracic pressure (Charlton *et al* 2017b). Thus, there is potential to extract much physiological information from the PPG signal.

The PPG signal can be acquired using a range of sensors and devices. PPG sensors consist of a light source such as a LED, and a light sensor, such as a photodiode (Sun and Thakor 2015). The light source illuminates a region with vasculature, such as the fingertip, and the light sensor measures how much light is either transmitted through or reflected from the tissue. In pulse oximeters, the PPG is typically acquired in transmission mode using a fingerclip probe (Nitzan *et al* 2020), while in consumer devices such as smartwatches, fitness trackers, and earbuds (i.e. *hearables*), it is typically acquired in reflection mode (Charlton and Marozas 2022). The amount of light received by sensors fluctuates with each heartbeat; usually, the amount of absorbed light increases during systole, when blood volume is greatest, and then decreases during diastole, when blood volume returns to its initial level (Allen 2007). This produces a pulse wave bearing several features, which can serve as physiological biomarkers (see figure 2) (Charlton *et al* 2022b).

Several factors can affect the morphology and quality of the PPG signal (Charlton *et al* 2022). First, PPG signals recorded during movement are often contaminated by motion artifacts (Park *et al* 2022). Second, PPG sensors must have good contact with the skin to obtain high-quality signals (Sun and Thakor 2015). Third, LED and photodiode positioning (Khan *et al* 2019), and acquiring multiple PPG signals (Charlton and Marozas 2022) can impact signal quality. Fourth, the wavelength of light emitted by the PPG sensor influences signal quality, with green wavelengths often preferred for reflectance mode PPG (Bashkatov *et al* 2005). Fifth, the contact pressure exerted by the sensor on the skin affects signal morphology (Chandrasekhar *et al* 2020). Sixth, the level of skin perfusion affects signal quality (Kyriacou *et al* 2002, Park *et al* 2022); low perfusion levels are usually associated with low wrist temperatures and with diseases such as Raynaud’s syndrome. Finally, the PPG measurement site (e.g. upper wrist) can affect signal morphology (Rajala *et al* 2018), quality (Prinable *et al* 2017) and susceptibility to motion artifact (Charlton *et al* 2022). Alternative sites, such as the arm, ear, chest, or face, may offer advantages in motion artifact reduction and in heart rate estimation accuracy (Charlton *et al* 2022). In addition, flexible and adhesive sensor attachments are emerging, promising improved contact and signal quality (Khan *et al* 2019).

### 1.2. Applications of photoplethysmography

At present, photoplethysmography is most commonly applied for heart rate monitoring in smartwatches (Temko 2017), and for oxygen saturation monitoring in pulse oximeters (Nitzan *et al* 2020). Smartwatches, fitness trackers and hearables are widely used, with an estimated 1.1 billion connected wearable devices worldwide in 2022 (Statista 2023). Pulse oximetry is a standard-of-care technique used in a range of clinical settings from intensive care to home monitoring (Ortega *et al* 2011, Greenhalgh *et al* 2021). Recently, the applications of photoplethysmography-based wearables have been expanded to include atrial fibrillation detection (Perez *et al* 2019), blood pressure monitoring (Vybornova *et al* 2021), and oxygen saturation monitoring (Spaccarotella *et al* 2022). Several additional potential applications of wearable photoplethysmography devices are being researched (Charlton *et al* 2023), including sleep staging (Kotzen *et al* 2022), mental health assessment (Cakmak *et al* 2021, Lyzwinski *et al* 2023), identifying obstructive sleep apnea (Behar *et al* 2014, 2019), and detection of peripheral arterial disease (Stansby *et al* 2022). Each of these applications uses PPG signal analysis to derive physiological information from the PPG.

Photoplethysmography confers several advantages over other physiological monitoring technologies, which have resulted in its widespread adoption. Measurements can be obtained quickly without the need for a trained operator, and photoplethysmography sensors are non-invasive, unobtrusive and low-cost. It is also a more compact and accessible monitoring modality than others, such as electrocardiogram (ECG) and blood pressure measurement. Furthermore, PPG measurements can be obtained without significantly disrupting daily activities, while ECG electrodes, for instance, can require careful placement and proper skin preparation to ensure accurate readings. However, a key disadvantage is that the PPG signal is highly susceptible to noise, such as in cases of poor sensor contact or motion (Li and Clifford 2012).

### 1.3. Standardising PPG signal analysis

A key step in the use of photoplethysmography for health and fitness monitoring is the development of PPG signal analysis algorithms. Such algorithms typically extract either inter-beat-intervals (e.g. for detection of atrial fibrillation) or PPG pulse wave shape features (e.g. for estimation of blood pressure). However, unlike in other application fields, such as heart rate variability analysis, there are no standards for PPG signal analysis, and only limited open tools are available. Consequently, standardized and reproducible analysis of PPG signals is lacking. Although there are some open-source PPG toolboxes, they lack validation and are often incomplete (see table 1).

**Table 1.** Comparison of open-source PPG signal processing toolboxes: pyPPG (this work), PPGFeat (Abdullah *et al* 2023), PulseAnalyse (Charlton *et al* 2019), NeuroKit2 (Makowski *et al* 2021), (RRest MIT Critical Data *et al* 2016), PPGSynth (Tang *et al* 2020), PhysioNet Cardiovascular Signal Toolbox (PCST) (Vest *et al* 2018), HeartPy (Van Gent *et al* 2019a, van Gent *et al* 2019b), BioSPPy (Carreiras *et al* 2015). Only toolboxes capable of prefiltering and detecting peaks in PPG signals are included.

	pyPPG	PPGFeat	PulseAnalyse	NeuroKit2	RRest	PPGSynth	PCST	HeartPy	BioSPPy
Prefiltering	✓	✓	✓	✓	✓	✓	✓	✓	✓
Peak detection	✓	✓	✓	✓	✓	✓	✓	✓	✓
Onset detection	✓	✓	✓	✓	✓	—	✓	—	✓
Other fiducial points	✓	✓	✓	—	—	—	—	—	—
Biomarker engineering	✓	—	✓	—	—	—	—	—	—
Signal quality	✓	—	✓	—	✓	✓	✓	✓	—
Quantitative validation	✓	✓	—	—	—	—	✓	✓	—
Programing language	Python	Matlab	Matlab	Python	Matlab	Matlab	Matlab	Python	Python

**Table 2.** Fingertip PPG databases used for the quantitative validation experiments.

Database	Number of subjects	Length of recordings	Gender (M:F)	Filtering	Sampling rate	Age
MESA	2056	∼10 h	1:1.2	Digital	256 Hz	54–95 years
PPG-BP	219	2 s	1:1.08	Hardware	1 kHz	20–89 years

Efficient analysis of long-term continuous physiological time-series poses a challenge for many PPG toolboxes. While a number of toolboxes enable accurate peak detection, they usually do not support the detection of other fiducial points and the engineering of standard digital biomarkers (see table 1). Moreover, a number of these toolboxes are implemented in Matlab, which limits their use by the wider open-source community. Although *PPGFeat* toolbox can detect most of the common PPG fiducial points, it is usable using a user interface that enables the analysis of a single pulse wave only.

Despite the extensive research and applications in the field of PPG analysis, there is an urgent need to standardize approaches, terminologies, variables and definitions. Furthermore, there is no comprehensive toolbox available that covers all standard PPG biomarkers. It is important to acknowledge that certain variables may have different terminologies in the scientific literature, but our objective was to unify them to facilitate a broader understanding of PPG biomarkers. To fill this gap, we developed standardized nomenclature and toolbox. The assigned names for variables aim to provide insights into their origin, while the definitions ensure accurate interpretation and improved comprehensibility.

### 1.4. Paper overview

**Figure 1.**
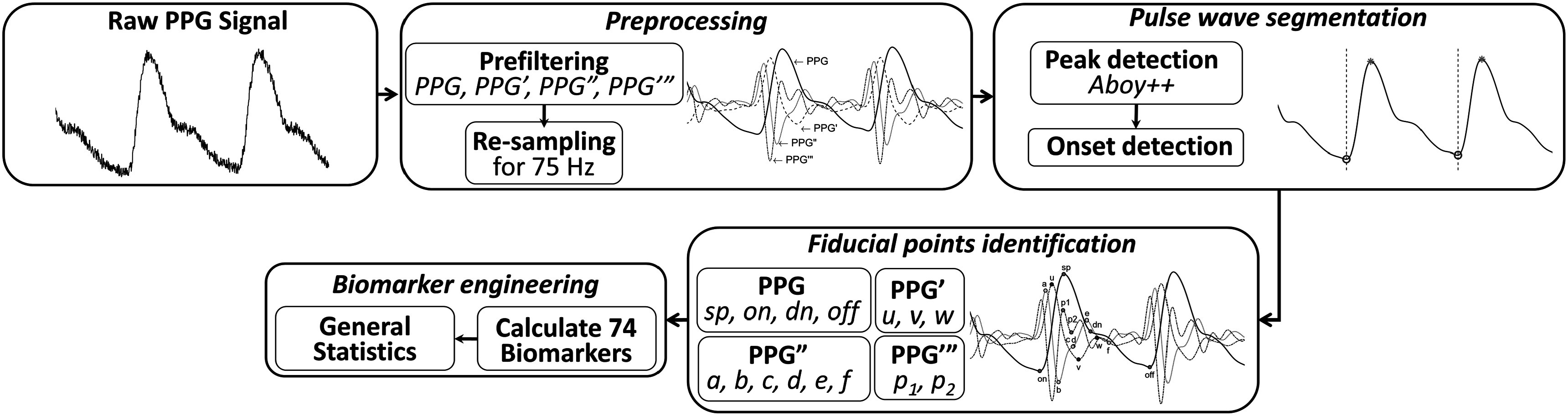
Flowchart for continuous PPG time series analysis. The terms PPG’, PPG”, and PPG‴ correspond to the first, second, and third derivatives of the PPG signal, respectively. The analysis comprises several key components, including: preprocessing, pulse wave segmentation, fiducial points identification and biomarker engineering.

The primary aim of this research was to create a standardized toolbox (*pyPPG*) for analysis if long-term finger PPG recordings in real-time. This paper presents standardized definitions for the state-of-the-art PPG fiducial points and biomarkers implemented within the *pyPPG* toolbox. It provides an overview of the steps involved in raw data processing and biomarker engineering, as well as validation of the fiducial point extraction process (see figure 1). Additionally, the paper presents performance results and benchmarks them against other publicly available toolboxes.

The *pyPPG* toolbox provides an open-source and validated fiducial point detection and extraction of standardized, state-of-the-art digital biomarkers for the continuous PPG time series.

## 2. Materials and methods

### 2.1. Databases

Two databases were used to validate the *pyPPG* toolbox (see table 2). The multi-ethnic study of atherosclerosis (MESA) database (Dean *et al* 2016, Zhang *et al* 2018) was used to validate the peak detector, and the PPG and blood pressure (PPG-BP) database (Liang *et al* 2018, Abdullah *et al* 2023) was used to validate the fiducial point detection algorithm. The MESA database consists of polysomnography (PSG) recordings from 2056 adults, aged 54–95 years, with subclinical cardiovascular disease, including 19 998 h of PPG recordings (Chen *et al* 2015, Rinkevičius *et al* 2023). Males accounted for 45.5% of the subjects. The database was downloaded from the National Sleep Resource Center (Zhang *et al* 2018). The PSG recordings in MESA were acquired at home, and included fingertip PPG signals measured at 256 Hz from the fingertip using Nonin 8000 series pulse oximeters (Nonin Medical Inc., Plymouth, US), alongside simultaneous ECG signals. The PPG-BP dataset contains 657 short (2 s) PPG recordings collected from 219 adult subjects, aged 20–89 years, with different health statuses (including healthy, hypertensive and diabetic subjects). Males accounted for 48% of the subjects. The data include fingertip PPG signals measured at 1 kHz using a SEP9AF-2 PPG sensor (SMPLUS Company, Korea). Signals were acquired using a 12 bit ADC, and the hardware applied a 0.512 Hz band-pass filter. Use of the retrospective databases available at open-access http://sleepdata.org for this research was approved by the institutional review board from the Technion-IIT Rappaport Faculty of Medicine (number 62-2019).

### 2.2. Overview of the pyPPG toolbox

The *pyPPG* toolbox is a standardized resource for real-time analysis of long-term finger PPG recordings. The toolbox consists of five main components, as summarized in figure 1:

1. **Loading a raw PPG signal:** the toolbox can accept various file formats such as .*mat*, .*csv*, .*txt*, or .*edf*. These files should contain raw PPG data along with the corresponding sampling rate.
2. **Preprocessing:** the raw PPG signal is filtered to remove noise and artifacts. Subsequently, the first, second, and third derivatives (PPG’, PPG”, and PPG”’) of the PPG signal are computed and filtered. The resampling of the filtered PPG signal to 75 Hz is specifically performed for systolic peak detection.
3. **Pulse wave segmentation:** the toolbox employs a peak detector to identify the systolic peaks. Based on the peak locations, the toolbox also detects the pulse onsets and offsets, which indicate the start and end of the PPG pulse waves.
4. **Fiducial points identification:** for each pulse wave, the toolbox detects a set of fiducial points.
5. **Biomarker engineering:** based on the fiducial points, a set of 74 PPG digital biomarkers are engineered.

The *pyPPG* toolbox also provides an optional PPG signal quality index based on the Matlab implementation of the work by Li and Clifford (2012). Signal quality assessment is performed based on template matching, which provides a numerical indicator for the quality of pulse waves (varying between 0 and 1). This can be used to identify distorted pulse waves. Whilst this signal quality index is provided by pyPPG, it was not used for the experiments conducted in this research.

### 2.3. Preprocessing

PPG signal filtering is one of the most essential parts of preprocessing. The human heart rate ranges between 30 and 200 beats per minute (Paliakaitė *et al* 2020). Therefore, in PPG signal analysis, it is common to apply bandpass filtering such 0.5−8 Hz (Abdullah *et al* 2023), 0.5−10 Hz (Finnegan *et al* 2023), 0.5−15 Hz (Mejia-Mejia *et al* 2022), 0.5−20 Hz (Allen and Murray 2000, Liang *et al* 2018), or 0.5−25 Hz (Chowdhury *et al* 2020), to conserve the frequency content of the PPG pulse waves while filtering out lower-frequency content (e.g. baseline wander due to respiration) and higher-frequency content (e.g. muscle noise or power interference).

**Figure A1.**
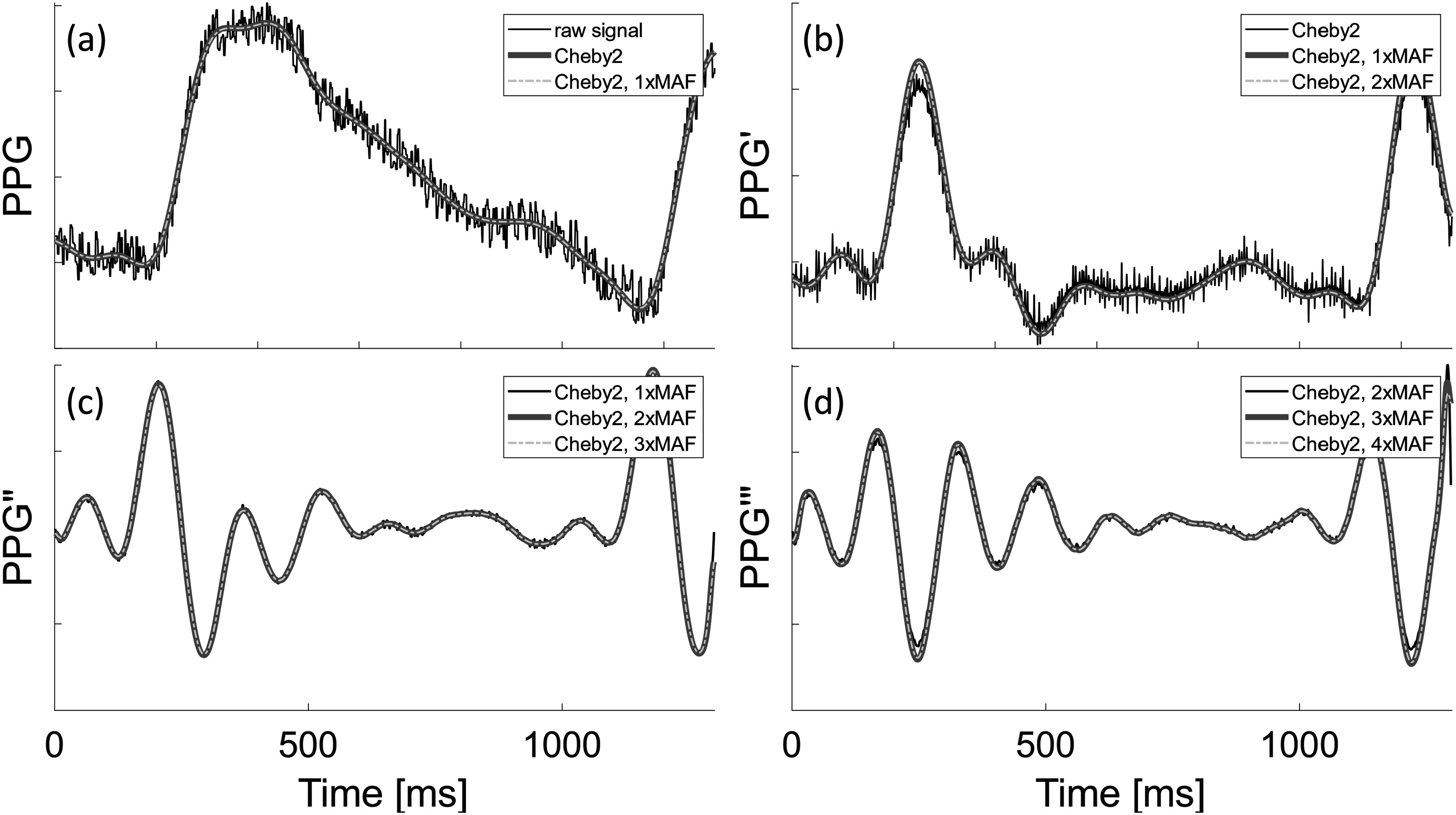
Prefiltering of the PPG, PPG, PPG, and PPG signals. In panel (a), the raw PPG signal is represented by the black curve, while the red curve represents the application of a fourth-order Chebyshev Type II filter (Cheby2). In panels (a)–(d), the green dashed curve represents the filtered version of the red curve obtained through moving average filtering (MAF). Panels (b)–(d) display the PPG’, PPG”, and PPG‴signals, respectively. The black curve depicts the derivatives of the red curve from the preceding panel. Likewise, the red curve corresponds to the derivative of the green curve shown in the previous panel.

Whilst fiducial point detection can be simpler with lower low-pass cut-off frequencies such as 8 Hz, the drawback of using lower cut-off frequencies is that they significantly distort the pulse wave shape and reduce the accuracy with which the pulse onset and other fiducial points can be identified. Conversely, cut-off frequencies above 12 Hz can make it more complex to detect fiducial points due to the presence of extra waves in the PPG derivatives. Therefore, during the benchmarking process of other toolboxes for fiducial point detection (see section 3.2), the 0.5−12 Hz frequency band was employed for filtering purposes. Although the 0.5−12 Hz band is recommended by default for PPG analysis, user can customize the passband filter in the *pyPPG* toolbox. The following zero-phase filters were implemented (see figure A1):

1. **Bandpass filtering between 0.5**−**12** **Hz:** a fourth-order Chebyshev Type II filter was used for the original signal. The 12 Hz low-pass cut-off filter was used to avoid time-shifting of fiducial points (particularly pulse onset, and dicrotic notch) and to eliminate unwanted high-frequency content from the PPG derivatives. The 0.5 Hz high-pass cut-off filter was used to minimize baseline wandering whilst retaining content at low heart rates.
2. **50 ms moving average filtering (MAF):** in the case of very noisy signals, some high-frequency content can remain in the band-pass filter signal. For this purpose, a 50 ms standard flat (boxcar or top-hat) MAF with a 9 Hz cut-off frequency was applied after the band-pass filtering.
3. **10 ms MAF for the PPG derivatives:** to eliminate the high-frequency content in the PPG derivatives, a 10 ms standard flat (boxcar or top-hat) MAF with 45 Hz cut-off frequency was applied.

The toolbox provides default filtering parameters that are optimized for fingertip PPG signals. Users have the flexibility to customize these parameters according to their requirements, e.g. cut-off frequencies, filter order and MAF size. The applied filtering technique minimally alters the amplitude, which is also verified on the PPG-BP dataset (see figure A1).

It is common for the PPG signal to be sampled at over 100 Hz and up to 1 kHz, as, for example, in the PPG-BP dataset. However, an excessive sampling frequency may not be ideal for long-term data processing due to the computational load. The default behavior of the toolbox is to resample PPG signals at 75 Hz using the Python *resample* function which is based on the Fourier method. Resampling was employed to reduce the computational load of the systolic peak detector. However, for the identification of other fiducial points the original sampling frequency was used.

### 2.4. Pulse wave segmentation

The toolbox identifies individual pulse waves in a PPG signal by identifying systolic peaks (*sp*), and then identifying the pulse onset (*on*) and offset (*off*) on either side of each systolic peak which indicate the start and end of the pulse wave, respectively.

#### 2.4.1. Systolic peak detection

**Figure 2.**
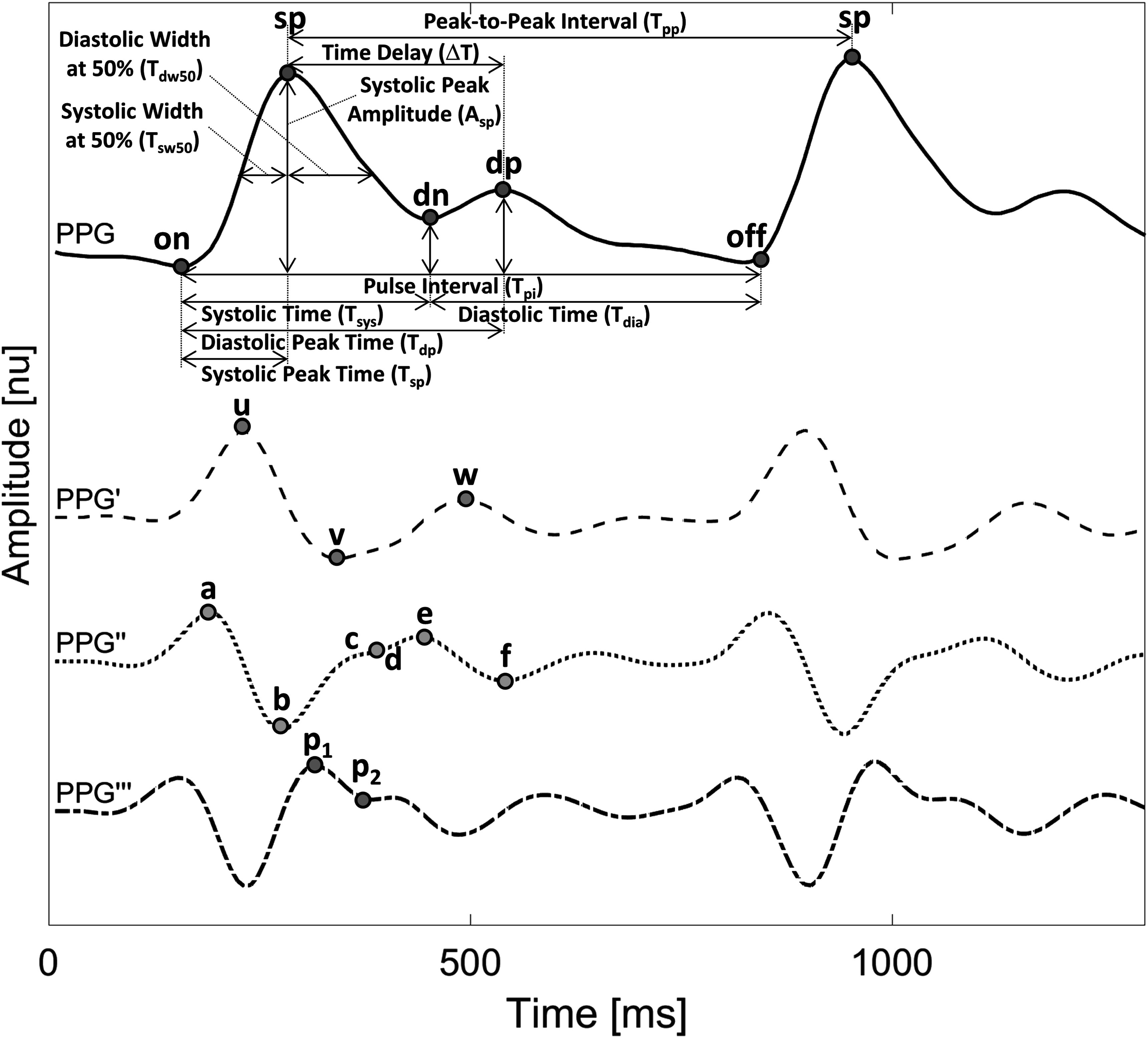
The fiducial points of the PPG signal include the systolic peak (*sp*), the pulse onset and offset (*on*, *off*), the dicrotic notch (*dn*) and the diastolic peak (*dp*). The fiducial points of PPG derivatives are represented by *u*, *v*, *w*, *a*, *b*, *c*, *d*, *e*, *f*, *p*_1_, *p*_2_. The biomarkers are calculated based on this set of fiducial points.

The *sp* is the most important fiducial point of the PPG signal (see figure 2). It is defined as the point with the highest amplitude between two consecutive pulse onsets (see figure 2). The *pyPPG* toolbox uses an enhanced *sp* detection algorithm to enable real-time analysis of long-term PPG measurements. The algorithm is an enhanced version of the Aboy beat detector (Aboy *et al* 2005), which performed either best (Kotzen 2022), or amongst the best (Charlton *et al* 2022a) in recent benchmarking studies of PPG beat detectors. We focused on improving the beat detector’s performance and reducing its computational complexity.

The original *Aboy* algorithm utilizes an advanced filtering technique to accurately detect systolic peaks (Aboy *et al* 2005). PPG recordings are segmented into 10-second windows and then filtered using three digital filters. The first filter helps to estimate the heart rate, while the second and third filters are used for peak detection. Two modifications were made to the *Aboy* algorithm (Aboy *et al* 2005). First, to enhance the speed of the previous Matlab implementation (Charlton *et al* 2022a), the finite impulse response (FIR) filter was replaced by a zero-phase fifth-order Chebyshev Type II infinite impulse response (IIR) filter, which applied the same cut-off frequencies as the original *Aboy* peak detector. Second, adaptive heart rate estimation is included to handle strong baseline wandering and rapid amplitude fluctuations (Goda *et al* 2023). When estimating heart rate, if the number of detected peaks is outside the expected lower and upper limits (see Goda *et al* 2023), then that 10 s segment of data is deemed to be low quality and the HR from the previous segment is retained. The resulting modified peak detector is denoted *Aboy++*.

When first presenting the *Aboy++* algorithm *et al* (Goda *et al* 2023), we evaluated it on a small subset consisting of 100 recordings from the MESA dataset containing over 4.25 million reference beats. *Aboy++* achieved an F1-score of 85.5% (79.78–92.57), compared to 80.99% (73.91–85.52) for the *Aboy* peak detector. In addition, the computational time of *Aboy++* was over 57 times faster than that of *Aboy*. Specifically, the median peak detection time for 1 h segments was 114.24 s for *Aboy*, compared to 1.98 s for *Aboy++*.

#### 2.4.2. Pulse onset detection

*On* corresponds to the beginning of the pulse wave and the beginning of the systolic upslope (see figure 2 and table 3). This systolic upslope is caused by increasing arterial pressure during systole (Addison 2016). *On* is typically, but not necessarily, a minimum point. *pyPPG* includes a novel *on* detection algorithm. Previously, *on* was identified as the minimum value between two successive detected *sp* (Farooq *et al* 2010, Vadrevu and Manikandan 2019), or identified using the slope sum function approach (Deshmane 2009, Nemati *et al* 2016). However, during long-term measurements there can be multiple local minima between successive *sp*, particularly in a noisy PPG signal. We define *on* as the initiation of the systolic upslope, which is usually a minimum point, although not always. We used a simple, yet accurate approach to detect *on* as the first maximum preceding the *p*_1_-point on the PPG‴. *off* is equivalent to *on* on the next pulse wave.

**Table 3.** Definition of PPG fiducial points.

Fiducial point definitions			References
**PPG**			
1	*on*	*Pulse onset*. The beginning of the systolic upslope, typically, but not necessarily, a minimum point	
2	*sp*	*Systolic peak*. The highest amplitude between two consecutive pulse onsets	
3	*dn*	*Dicrotic notch*. If a diastolic peak is present, then it is the local minimum preceding the diastolic peak. If there is no diastolic peak, then it is the inflection point between the systolic peak and f-point
4	*dp*	*Diastolic peak*. The first local maximum of the PPG pulse wave after the dicrotic notch and before the 0.8 pulse interval; if there is no maxima, then the first local maximum of the PPG pulse wave after the e-point and before the 0.8 pulse interval.	Takazawa *et al* (1998)
5	*off*	*Pulse offset*. The local minimum preceding the next pulse wave’s systolic upslope

**PPG′**			
5	*u*	The highest amplitude between the pulse onset and systolic peak on PPG′	Alty *et al* (2003)
6	*v*	The lowest amplitude between the u-point and diastolic peak on PPG′	Suboh *et al* (2022)
7	*w*	The first local maximum or inflection point after the dicrotic notch on PPG	Suboh *et al* (2022)

**PPG″**			
8	*a*	The highest amplitude between pulse onset and systolic peak on PPG″	Takazawa *et al* (1998)
9	*b*	The first local minimum after the a-point on PPG″	Takazawa *et al* (1998)
10	*c*	The local maximum with the highest amplitude between the b-point and e-point, or if no local maximum is present, then the inflection point on PPG″	Takazawa *et al* (1998)
11	*d*	The local minimum with the lowest amplitude between the c-point and e-point, or if no local minimum is present, then the inflection point on PPG″	Takazawa *et al* (1998)
12	*e*	The local maximum with the highest amplitude after the b-point and before the diastolic peak on PPG″	Takazawa *et al* (1998)
13	*f*	The first local minimum after the e-point on PPG″	Takazawa *et al* (1998)

**PPG**‴			
14	*p*_1_	The first local maximum after the b-point on PPG‴	Charlton *et al* (2018)
15	*p*_2_	The last local minimum after the b-point and before the d-point on PPG‴	

### 2.5. Fiducial points detection

Table 4 summarises the algorithmic approaches used to detect fiducial points. The approaches were designed based on those used in *PulseAnalyse*. Additional approaches were created for the fiducial points which were not implemented in *PulseAnalyse* (for *v* and *w* points). The approaches were then refined according to the fiducial point definitions presented in table 3.

**Table 4.** Detection and correction of PPG fiducial points.

Fiducial point detections and corrections	
*sp*	Initially detected based on *Aboy++* algorithm (2023), and corrects the peaks’ location (interbeat intervals) error.

	Initially detected based on following rules:
*on*,	(1) minimum point before *sp*
	(2) successive onsets Followed by the first maximum preceding the *p*_1_-point on the PPG‴
*off*	(3) *T*_*sp*_ ^a^ is minimum 120 ms
	I (4) *T*_*dp*_ ^b^ is minimum 300 ms

*dn*	Initially detected based on Balmer’s algorithm (Balmer *et al* 2018), followed by searching for a minimum point between the *sp* and *dp*. If the time difference exceeds 100 ms between the identified *dn* and the minimum point, the value of *dn* is then substituted with the identified minimum point. (This correction was not used for PPG-BP dataset)

*dp*	Initially detected based on the definition in table 3, followed by recalculate based on definition using updated values of *on*, *dn*, *e* and *off*. (This correction was not used for PPG-BP dataset)

*v*	Initially detected based on the definition in table 3, followed by the *v* is local minimum between *u* and *e*.

*w*	Initially detected based on the definition in table 3, followed by the *w* is local maximum between *e* and *f*, following the *v*.

*e*	Detected based on definition, with the additional constraint of an upper bound of 60% of the pulse wave duration.

*f*	Detected based on the definition in table 3, followed by recalculate using updated value of *w* and an additional constraint of an upper bound of 80% of the pulse wave duration.

*p*_2_	Detected based on *c*, *d*, *e* points.

^a^ Systolic time, the time between the pulse onset and dicrotic notch.

^b^ Diastolic time, the time between the dicrotic notch and pulse offset. In the case of *u*, *a* − *d* and *p*_1_, the detection proceeds the definition according to table 3.

#### 2.5.1. Dicrotic notch detection

The dicrotic notch (*dn*) plays an important role as a fiducial point in the analysis of PPG signals, holding immense potential for various applications such as heart disease detection (Gu *et al* 2008) and arterial stiffness assessment (Addison 2016). Its significance stems from its association with the duration of systole, which is known to be affected by heart disease. Additionally, appearance of the diastolic wave following the *dn* allows for the evaluation of arterial stiffness, with the hypothesis that the presence of the *dn* is influenced by the arterial stiffness. However, it should be noted that the visibility of the *dn* diminishes progressively with age, making it typically no longer discernible in elderly subjects (Charlton *et al* 2022c).

**Figure 3.**
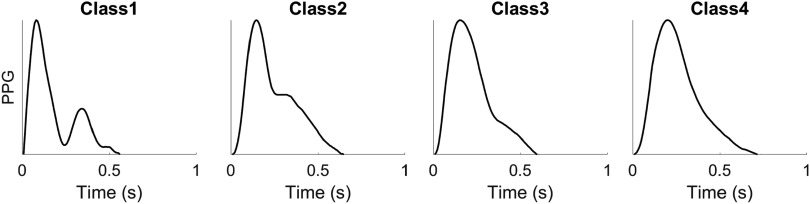
Different classes of PPG pulse waves according to the characteristics of the dicrotic notch, as defined by Dawber *et al* (1973). The figure was adapted from Charlton *et al* (2022c).

None of the existing definitions of *dn* are entirely satisfactory. Typically, the *dn* is easily recognizable when a distinct local minimum exists between the *sp* and the *dp* (see figure 2). Yet, in many cases, the *dp* is not clearly visible, rendering it difficult to accurately identify the *dn*. Dawber *et al* (1973) categorized different classes of *dn*, which are illustrated in figure 3. The fiducial point *dn* can be classified into four classes: Class 1, in which the *dn* is an incisura, Class 2, in which there is a horizontal line at the *dn*, Class 3, in which there is a change in gradient on the downslope, and Class 4, in which there is no clear evidence of the *dn*.

Another morphological approach for *dn* identification involves locating it at the time of zero-crossing of the PPG” between the *d* and *e* points (Chakraborty *et al* 2021). Yet, situations may arise where the local minimum of the *dn* is visible, but the occurrence of the *d* and *e* points precedes the zero-crossing point, as depicted in figure 2. Thus, debate regarding the precise location for defining the *dn* is ongoing.

#### 2.5.2. Fiducial points of PPG derivatives

Additional fiducial points were defined on the PPG derivatives (PPG’, PPG” and PPG”’) as depicted in figure 2 (Charlton *et al* 2022c, Suboh *et al* 2022). The fiducial point detection algorithms in *pyPPG* are based on standardized, morphological definitions (see table 3). Consequently, these points do not necessarily correspond to points with consistent physiological interpretations. On the PPG’ signal, the maximum point of the systolic slope is denoted as the *u*-point, while the minimum point is referred to as the *v*-point. The *u*-point has been used to assess arterial stiffness (von Wowern *et al* 2015). On the PPG” signal, six further fiducial points are defined. Among these, four points (*a*, *b*, *c*, and *d*) are typically observed during the systolic phase (see figure 2). As the diastolic phase begins, the *e*-point becomes visible, followed by the appearance of the *f*-point (Suboh *et al* 2022). Points *a* to *e* have been used to assess vascular ageing (Takazawa *et al* 1998), and the *d*-point has been identified as a predictor of cardiovascular mortality. On the PPG”’ signal, *p*_1_ represents the early systolic component of the PPG pulse wave, while *p*_2_ corresponds to the late systolic component (Takazawa *et al* 1998). *p*_1_ and *p*_2_ are used to calculate the augmentation index, which has been found to be elevated in atherosclerotic and diabetic subjects (Bortolotto *et al* 2000, Pilt *et al* 2014).

#### 2.5.3. Correction of the fiducial points

After *pyPPG* detects the fiducial points, some postprocessing routines are applied (see table 4). Each fiducial point is represented only once per pulse wave. In the correction process, multiple fiducial points are utilized to ascertain the accurate positioning of fiducial points based on their expected relative position. Inconsistent points are adjusted according to other fiducial points or discarded. This postprocessing routine is optional and can be disabled by the user.

### 2.6. Biomarker engineering

We implemented standardized biomarkers, employing uniform definitions for annotation. While the *PulseAnalyse* toolbox provides important biomarkers, *pyPPG* not only includes a more extensive collection of biomarkers but also features their up-to-date implementation. More specifically, the *pyPPG* toolbox includes a comprehensive collection of 74 standard PPG morphological biomarkers which are calculated from the timings and amplitudes of the fiducial points (see tables 5–8). The biomarkers were categorized into four groups: (1) PPG Signal—biomarkers that are based on the location of the fiducial points of the PPG signal, (2) Signal Ratios—biomarkers that are based on ratios of the fiducial points of the PPG signal, (3) PPG Derivatives—biomarkers that are based on the location of the fiducial points of the PPG derivatives and (4) Derivatives Ratios—biomarkers that are based on ratios of the fiducial points of the PPG derivatives.

**Table 5.** Biomarkers derived from the PPG signal categorized by intervals, amplitudes and areas.

PPG Signal				References
1	Intervals	***T***_*pi*_	Pulse interval, the time between the pulse onset and pulse offset	Chowdhury *et al* (2020)
2		***T***_*pp*_	Peak-to-peak interval, the time between two consecutive systolic peaks	Kurylyak *et al* (2013)
3		***T***_*sys*_	Systolic time, the time between the pulse onset and dicrotic notch	Ahn (2017)
4		***T***_*dia*_	Diastolic time, the time between the dicrotic notch and pulse offset	Ahn (2017)
5		***T***_*sp*_	Systolic peak time, the time between the pulse onset and systolic peak	Alty *et al* (2003)
6		***T***_*dp*_	Diastolic peak time, the time between the pulse onset and diastolic peak	Chowienczyk *et al* (1999)
7		Δ ***T***	Time delay, the time between the systolic peak and diastolic peak	Chowienczyk *et al* (1999)
8		***T***_*swx*_	Systolic width, the width at x% of the systolic peak amplitude between the pulse onset and systolic peak	Kurylyak *et al* (2013)
9		***T***_*dwx*_	Diastolic width, the width at x% of the systolic peak amplitude between the systolic peak and pulse offset	Kurylyak *et al* (2013)
10		***T***_*pwx*_	Pulse width, the sum of the systolic width and diastolic width at x%	Kurylyak *et al* (2013)

11	Amplitudes	***A***_*sp*_	Systolic peak amplitude, the difference in amplitude between the pulse onset and systolic peak	Chua and Heneghan (2006)
12		${{\boldsymbol{A}}}_{{dn}}$	Dicrotic notch amplitude, the difference in amplitude between the pulse onset and dicrotic notch	Duan *et al* (2016)
13		***A***_*dp*_	Diastolic peak amplitude, the difference in amplitude between the pulse onset and diastolic peak	Duan *et al* (2016)
14		***A***_*off*_	Pulse onset amplitude, the difference in amplitude between the pulse onset and pulse offset	

15	Areas	***AUC***_*pi*_	Area under pulse interval curve, the area under the pulse wave between pulse onset and pulse offset	Duan *et al* (2016)
16		***AUC***_*sys*_	Area under systolic curve, the area under the pulse wave between the pulse onset and dicrotic notch	Ahn (2017)
17		***AUC***_*dia*_	Area under diastolic curve, the area under the pulse wave between the dicrotic notch and pulse offset	Ahn (2017)

**Table 6.** Biomarkers derived from the signal ratios categorized by intervals, amplitudes, areas and combinations thereof.

Signal ratios				References
1	Intervals	*IPR*	Instantaneous pulse rate, 60/T_*pi*_	Lueken *et al* (2017)
2		***T***_*sys*_/***T***_*dia*_	Ratio of the systolic time versus the diastolic time	Ahn (2017)
3		***T***_*pwx*_/***T***_*pi*_	Ratio of the pulse width at x% of the systolic peak amplitude versus the pulse interval	Chowdhury *et al* (2020)
4		***T***_*pwx*_/***T***_*ps*_	Ratio of the pulse width at x% of the systolic peak amplitude versus the systolic peak time	Chowdhury *et al* (2020)
5		***T***_*dwx*_/***T***_*swx*_	Ratio of the diastolic width versus the systolic width at x% width	Kurylyak *et al* (2013)
6		***T***_*sp*_/***T***_*pi*_	Ratio of the systolic peak time versus the pulse interval	

7	Amplitudes	***A***_*sp*_/*A*_*off*_	Ratio of the systolic peak amplitude versus the pulse offset amplitude	
8		***A***_*dp*_/*A*_*sp*_	Reflection index, ratio of the diastolic peak amplitude versus the systolic peak amplitude	Chowienczyk *et al* (1999)

9	Areas	*IPA*	Inflection point area, ratio of the area under diastolic curve versus the area under systolic curve	Wang *et al* (2009)

10	Combined	***T***_*sp*_/*A*_*sp*_	Ratio of the systolic peak time versus the systolic peak amplitude	Liu *et al* (2021)
11		***A***_*sp*_/Δ ***T***	Stiffness index, ratio of the systolic peak amplitude versus the time delay	Millasseau *et al* (2002)
12		***A***_*sp*_/(***T***_*pi*_ − ***T***_*sp*_)	Ratio of the systolic peak amplitude versus the difference between the pulse interval and systolic peak time	Chowdhury *et al* (2020)

**Table 7.** Biomarkers derived from the PPG derivatives.

PPG derivatives				References
1	Intervals	***T***_*u*_	u-point time, the time between the pulse onset and u-point	ms
2		***T***_*u*_	v-point time, the time between the pulse onset and v-point	Suboh *et al* (2022)
3		***T***_*w*_	w-point time, the time between the pulse onset and w-point	Suboh *et al* (2022)
4		***T***_*a*_	a-point time, the time between the pulse onset and a-point	Suboh *et al* (2022)
5		***T***_*b*_	b-point time, the time between the pulse onset and b-point	Suboh *et al* (2022)
6		***T***_*c*_	c-point time, the time between the pulse onset and c-point	Suboh *et al* (2022)
7		***T***_*d*_	d-point time, the time between the pulse onset and d-point	Suboh *et al* (2022)
8		***T***_*e*_	e-point time, the time between the pulse onset and e-point	Suboh *et al* (2022)
9		***T***_*f*_	f-point time, the time between the pulse onset and f-point	Suboh *et al* (2022)
10		***T***_*b*−*c*_	b−c time, the time between the b-point and c-point	Charlton *et al* (2018)
11		***T***_*b*−*d*_	b−d time, the time between the b-point and d-point	Charlton *et al* (2018)
12		${{\boldsymbol{T}}}_{{p}_{1}}$	p_1_-point time, the time between the pulse onset and p_1_-point	Suboh *et al* (2022)
13		${{\boldsymbol{T}}}_{{p}_{2}}$	p_2_-point time, the time between the pulse onset and p_2_-point	Suboh *et al* (2022)
14		${{\boldsymbol{T}}}_{{p}_{1}-{dp}}$	p_1_ − dia time, the time between the p_1_-point and diastolic peak	Peltokangas *et al* (2017)
15		${{\boldsymbol{T}}}_{{p}_{2}-{dp}}$	p_2_ − dia time, the time between the p_2_-point and diastolic peak	Peltokangas *et al* (2017)

**Table 8.** Biomarkers derived from the derivatives ratios categorized into intervals, amplitudes, areas, and combinations of these.

Derivatives ratios				References
1	Intervals	*Tu/Tpi*	Ratio of the u-point time versus the pulse interval	Chowdhury *et al* (2020)
2		***T***_*v*_/***T***_*pi*_	Ratio of the v-point time versus the pulse interval	Chowdhury *et al* (2020)
3		***T***_*w*_/***T***_*pi*_	Ratio of the w-point time versus the pulse interval	
4		***T***_*a*_/***T***_*pi*_	Ratio of the a-point time versus the pulse interval	Chowdhury *et al* (2020)
5		***T***_*b*_/***T***_*pi*_	Ratio of the b-point time versus the pulse interval	Chowdhury *et al* (2020)
6		***T***_*c*_/***T***_*pi*_	Ratio of the c-point time versus the pulse interval	
7		***T***_*d*_/***T***_*pi*_	Ratio of the d-point time versus the pulse interval	
8		***T***_*e*_/***T***_*pi*_	Ratio of the e-point time versus the pulse interval	
9		***T***_*f*_/***T***_*pi*_	Ratio of the f-point time versus the pulse interval	
10		(***T***_*u*_ − ***T***_*a*_)/***T***_*pi*_	Ratio of the difference between the u-point-time and a-point time versus the pulse interval	Chowdhury *et al* (2020)
11		(***T***_*v*_ − ***T***_*b*_)/***T***_*pi*_	Ratio of the difference between the v-point time and b-point time versus the pulse interval	Chowdhury *et al* (2020)

12	Amplitudes	***A***_*u*_/*A*_*sp*_	Ratio of the u-point amplitude versus the systolic peak amplitude	Alty *et al* (2003)
13		***A***_*v*_/*A*_*u*_	Ratio of the v-point amplitude versus the u-point amplitude	
14		***A***_*w*_/*A*_*u*_	Ratio of the w-point amplitude versus the u-point amplitude	
15		***A***_*b*_/*A*_*a*_	Ratio of the b-point amplitude versus the a-point amplitude	Takazawa *et al* (1998)
16		***A***_*c*_/*A*_*a*_	Ratio of the c-point amplitude versus the a-point amplitude	Takazawa *et al* (1998)
17		***A***_*d*_/*A*_*a*_	Ratio of the d-point amplitude versus the a-point amplitude	Takazawa *et al* (1998)
18		***A***_*e*_/*A*_*a*_	Ratio of the e-point amplitude versus the a-point amplitude	Takazawa *et al* (1998)
19		***A***_*f*_/*A*_*a*_	Ratio of the f-point amplitude versus the a-point amplitude	
20		${{\boldsymbol{A}}}_{{p}_{2}}/{{\boldsymbol{A}}}_{{p}_{1}}$	Ratio of the p_2_-point amplitude versus the p_1_-point amplitude	Peltokangas *et al* (2017)
21		(***A***_*c*_ − *A*_*b*_)/*A*_*a*_	Ratio of the difference between the b-point amplitude and c-point amplitude versus the a-point amplitude	Ahn (2017)
22		(***A***_*d*_ − ***A***_*b*_)/***A***	Ratio of the difference between the b-point amplitude and d-point amplitude versus the a-point amplitude	Ahn (2017)
23		***AGI***	Aging index, (A_*b*_-A_*c*_-A_*d*_-A_*e*_)/A_*a*_	Takazawa *et al* (1998)
24		${{\boldsymbol{AGI}}}_{{mod}}$	Modified aging index, (A_*b*_-A_*c*_-A_*d*_)/A_*a*_	Ushiroyama *et al* (2005)
25		${{\boldsymbol{AGI}}}_{{\inf }}$	Informal aging index, (A_*b*_-A_*e*_)/A_*a*_	Baek *et al* (2007)
26		*AI*	Augmentation index, (PPG(Tp2)-PPG(Tp1))/Asp	Takazawa *et al* (1998)
27		${{\boldsymbol{RI}}}_{{p}_{1}}$	Reflection index of p_1_, A_*dp*_/(PPG(T${}_{{p}_{1}}$)-PPG(T_*pi*_(0)))	Peltokangas *et al* (2017)
28		${{\boldsymbol{RI}}}_{{p}_{2}}$	Reflection index of p_2_, A_*dp*_/(PPG(p_2_)-PPG(T_*pi*_(0)))	Peltokangas *et al* (2017)

29	Combined	***SC***	Spring constant, PPG”(T_*sp*_)/((A_*sp*_-A_*u*_)/A_*sp*_)	Wei (2013)
30		***IPAD***	Inflection point area plus normalised d-point amplitude, AUC_*dia*_/AUC_*sys*_+A_*d*_/A_*a*_	Ahn (2017)

For a given window consisting of a set of beats, *pyPPG* provides the following nine general statistics for each biomarker (see appendix tables A2–A5): average (AVG), median (MED), standard deviation (SD), lower and upper quartiles (Q1, Q3), inter-quartile range (IQR), skewness (SKW, indicating a lack of symmetry in the distribution), kurtosis (KUR, indicating the pointedness of a peak in the distribution curve), and the average difference between the mean and each data value (MAD). For each biomarker, we provide these summary statistics including measures of the central tendency and dispersion.

### 2.7. Validation

#### 2.7.1. Systolic peak detection

The performance and computational complexity of *Aboy++* were evaluated. Performance was assessed in comparison to reference ECG-derived beats using the *F*_1_-score, which is a commonly used statistic for evaluating the performance of such algorithms. The *F*_1_-score is particularly suitable for this purpose because it effectively combines multiple fractional measures by utilizing a harmonic mean between the sensitivity and positive predictive value. *F*_1_-scores are reported as MED and quartiles (Q1, Q3). The performance and computational complexity of *Aboy++* were compared to against the implementation of *Aboy* provided by Charlton *et al* (2022a). Due to the high computational needs of *Aboy*, the two algorithms were compared on a subset of MESA consisting of 100 PPG recordings (1173 h). *Aboy++* was then assessed on the entire MESA database, with the exception of two recordings which did not have an ECG reference signal. Thus 2054 PPG recordings, consisting of more than 19 000 h of continuous PPG signals and over 91 million reference beats were included. The median recording length was 10 h, with a 2.5 h interquartile range (IQR). The 10 h long recordings were divided into 10 min segments. Segments were excluded if they did not contain a minimum of 300 ECG reference beats or if the extracted biomarkers could not be successfully evaluated. A key step in this assessment was to synchronise the timings of ECG-derived beats and PPG systolic peaks. This was achieved by forecasting the PPG *sp* by extracting electrocardiogram (ECG) peaks from the PSG recordings as a reference signal, similar to the work of Kotzen *et al* (2021). The evaluation metric was based on the alignment of the ECG-R-wave and PPG *sp*. The methods for performance assessment are elaborated in our previous work (Kotzen *et al* 2021, Goda *et al* 2023).

#### 2.7.2. Fiducial point detection

The fiducial point detection algorithm was validated by comparison against the manual annotations of the PPG-BP (Liang *et al* 2018) database. The data were manually annotated by two annotators (MG and PC) per the definitions in table 3. An annotation tool was adapted from the open source RRest toolbox for this purpose (Charlton *et al* 2017b). Fiducial points that could not be confidently identified were not annotated it. After both annotators independently annotated the prefiltered signal, the inter-annotator time differences were calculated. If the time discrepancy was >10 ms, then the annotators discussed the case and either agreed on a location or excluded the fiducial point (i.e. the annotators were not confident of its location). The final reference annotations were determined as the average of the annotations provided by the two annotators. In the PPG-BP database, each subject has three recordings. The first complete, high-quality pulse wave was selected for each subject. In total, more than 3000 fiducial points from 219 patients were manually annotated by the two annotators. The PPG-BP dataset typically includes high-quality pulse waves. However, for the final evaluation, certain fiducial points were excluded due to unclear or ambiguous annotations (see table 9). Only 1 element (<1%) was excluded for *w*, 2 elements (∼1%) for *p*_2_, 7 elements combined for *c* and *d* (∼3%), and 29 elements (∼13%) for *dn* out of a total of 219. We provide access to the manual annotations on the https://pyppg.readthedocs.io website. The PPG signals were filtered with a 12 Hz cut-off frequency during the manual annotation. The inter-annotator reliability of annotations is presented in table A1.

**Table 9.** Benchmark of PPG toolboxes for the detection of 219 fiducial points.

Fiducial point	*sp*	*on*	*dn*	*u*	*v*	*w*	*a*	*b*	*c*	*d*	*e*	*f*	*p*_*1*_	*p*_*2*_
No. excluded points^a^	0	0	29	0	0	1	0	0	7	7	0	0	0	2
*pyPPG (this work)*	**5(9)**	**7(10)**	**9(12)**	**1(1)**	**2(7)**	**3(6)**	**1(1)**	**2(2)**	**4(6)**	**4(6)**	**2(2)**	**3(8)**	**1(1)**	**2(3)**
*PulseAnalyse* Charlton *et al* (2019)	**5(9)**	13(37)	24(18)	2(1)	—	—	**1(1)**	**2(2)**	9(27)	9(26)	3(15)	5(19)	37(35)	28(40)
*PPGFeat* Abdullah *et al* (2023)	26(24)	**7(27)**	50(47)	16(44)	15(21)	53(99)	19(38)	17(37)	22(40)	26(38)	28(46)	37(65)	—	—

^a^ This number refers to fiducial points that have been excluded due to unclear or ambiguous annotations. The mean (and standard deviation) of the absolute errors are reported for each fiducial in ms.

#### 2.7.3. Fiducial point benchmarking

To assess the performance of the fiducial point detection algorithm, *pyPPG* was benchmarked against two publicly available PPG toolboxes capable of detecting fiducial points (*PulseAnalyse* (Charlton *et al* 2019) and *PPGFeat* (Abdullah *et al* 2023)). Both benchmarked toolboxes were implemented in Matlab. Performance was assessed using the mean absolute error (MAE) and the standard deviation of the absolute errors (SD) of the fiducial point detections in comparison to the reference. Bland–Altman plots (Bland and Altman 1986) with the limits of agreement (1.96SD, indicating 95% of errors) are also provided.

In benchmarking, the same reference labels were used. For all toolboxes, the PPG signals were filtered between 0.5 and 12 Hz. In the case of *pyPPG* and *PPGFeat*, PPG signals were filtered using the built-in filtering capabilities. However, for *PulseAnalyse*, we provided pre-filtered data as input to the toolbox as this toolbox does not enable the pre-filtering of short PPG segments.

## 3. Results

### 3.1. pyPPG peak detection

The *pyPPG* peak detector was evaluated on the 2054 recordings of the MESA dataset, which included more than 91 million reference beats. The peak detection achieved a median *F*_1_-score of 88.19% (lower—upper quartiles of 81.73%–92.71%). *pyPPG* demonstrated the same performance in real-time operation.

### 3.2. Evaluation of benchmarking

**Figure 4.**
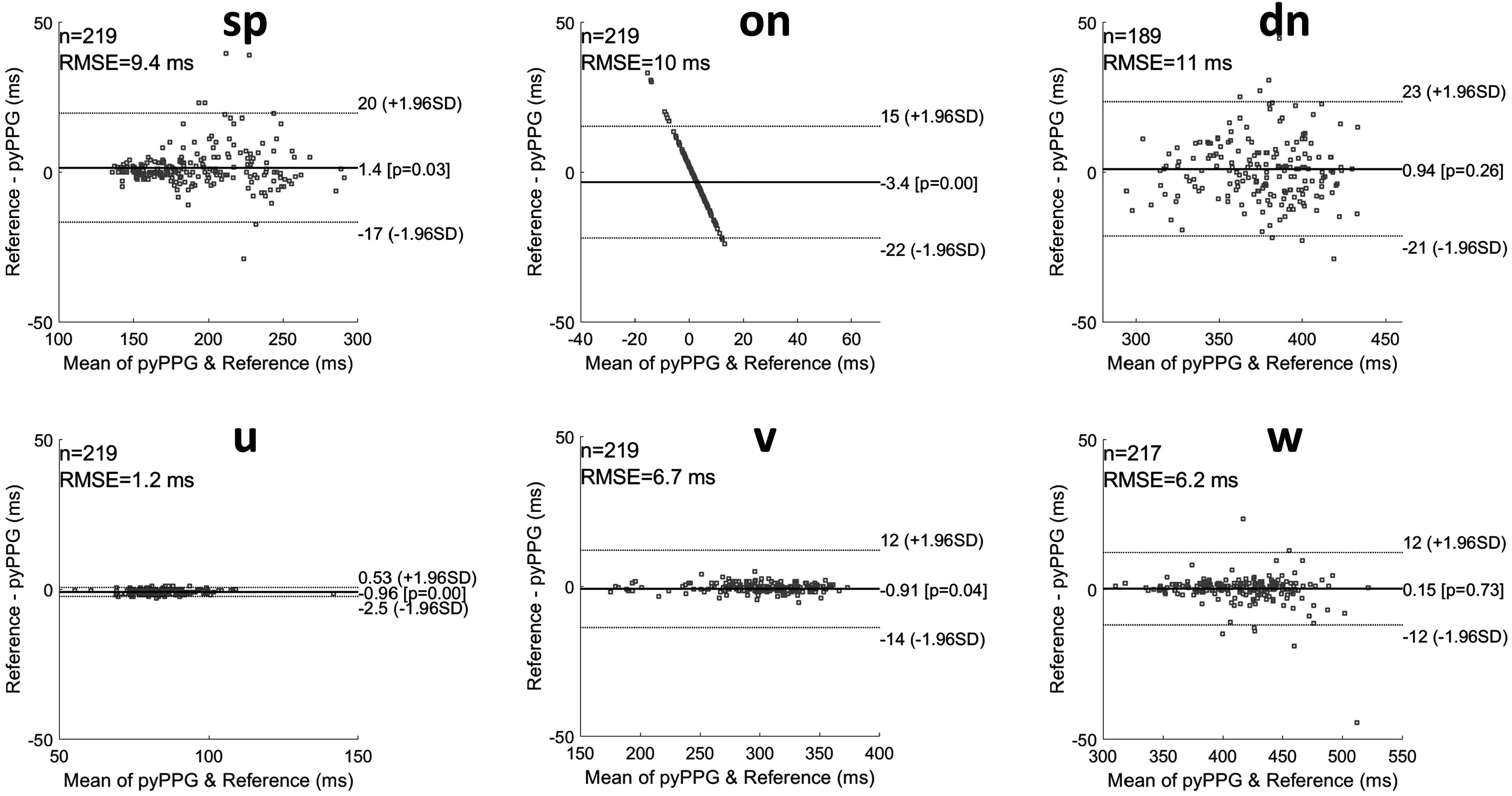
Bland–Altman plots for fiducial points of PPG and PPG’. RMSE: root mean squared error, *n*: number of fiducial points, ±1.96SD: the limits of agreement, and Pearson correlation *p*-value.

**Figure 5.**
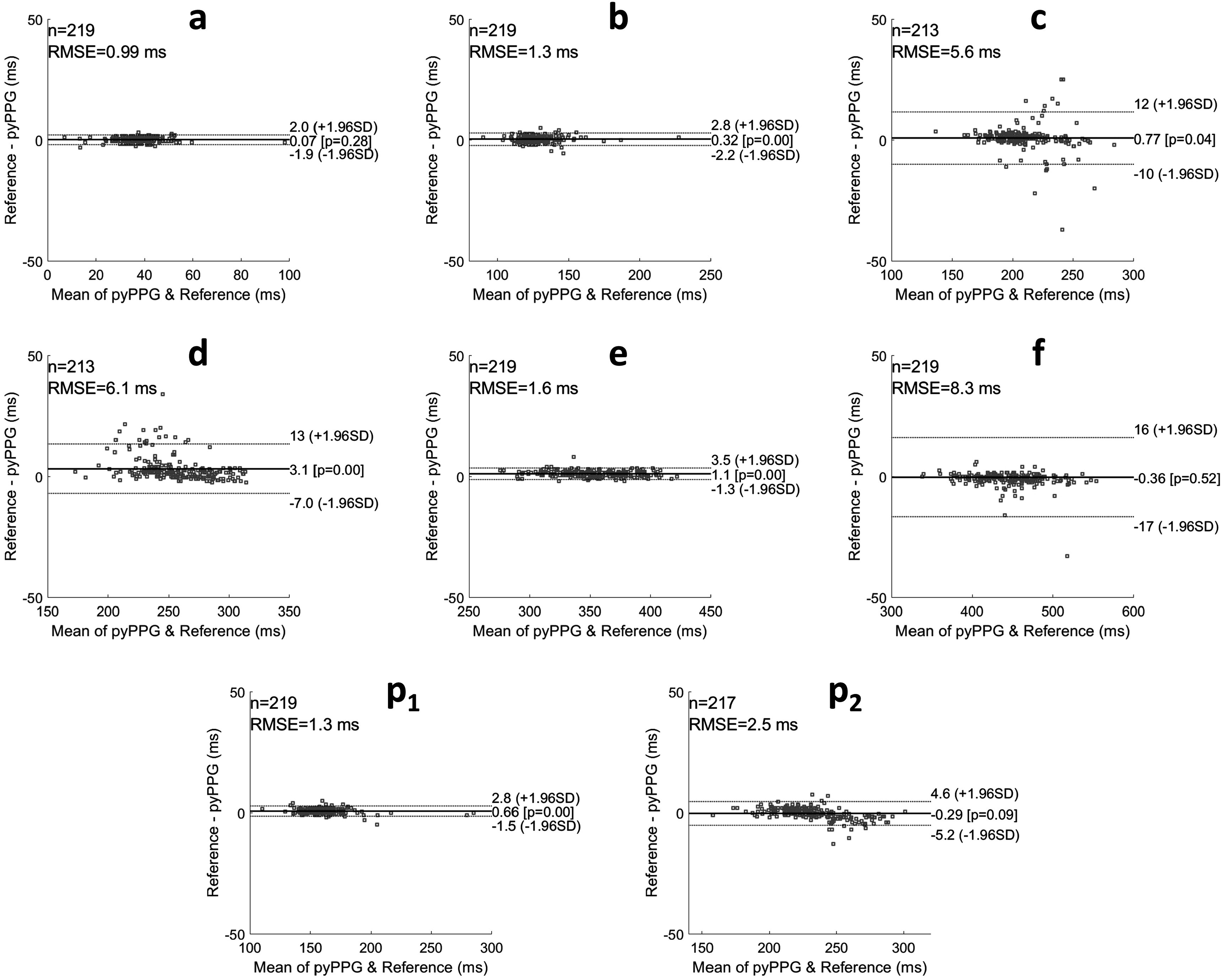
Bland–Altman plots for fiducial points of PPG” and PPG‴. RMSE: root mean squared error, *n*: number of fiducial points, ±1.96SD: the limits of agreement, and Pearson correlation *p*-value.

**Figure A2.**
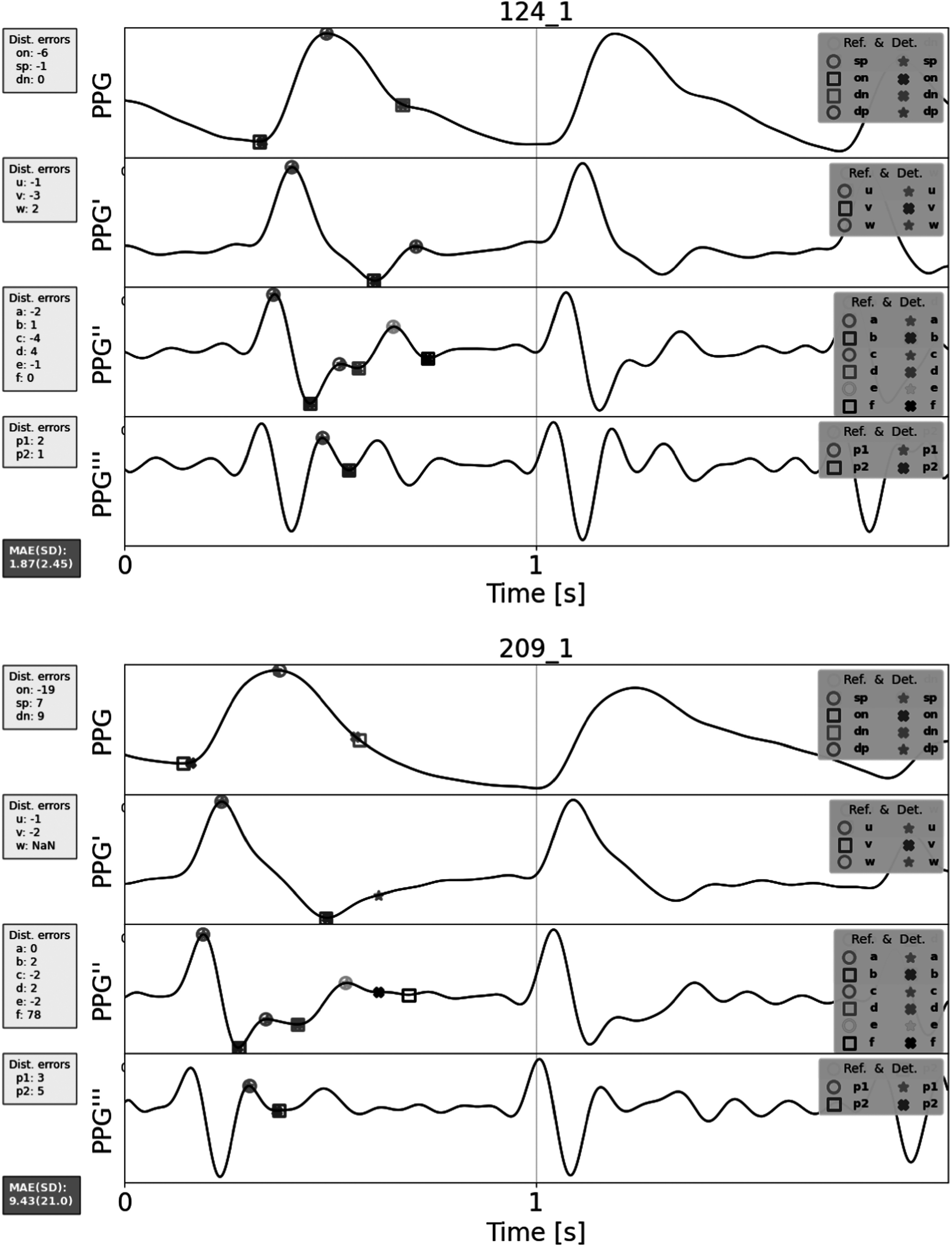
A comparison between high and low-quality PPG’ and PPG” signals. Record 124_1 is a high-quality signal, where all fiducial points are easily detectable. Conversely, in the case of Record 209_1, the annotation of fiducial points *w* and *f* was unclear or ambiguous. The reference fiducial points are represented by empty circles and squares, while the detected points are indicated by crosses and stars.

The results for the benchmarking of *pyPPG* against other PPG toolboxes (*Pulse* *Analyse* and *PPGFeat*) are presented in table 9. A total of 219 distinct pulse waves were employed for the benchmarking process. With *pyPPG*, the MAEs were <10 ms for all fiducial points, were less than one fifth of those for *PPGFeat* for all fiducial points except *on*, and less than those for *PulseAnalyse* for all except three fiducial points (*sp*, *a*, *b*). In comparison to the other toolboxes, *pyPPG* showed particular improvements in the detection of *dn*, *p*_1_ and *p*_2_. In addition, *pyPPG* was able to detect fiducial points (see figure A2) which were not detected by *PulseAnalyse* (*v* and *w*) or *PPGFeat* (*p*_1_, *p*_2_). Bland–Altman plots were generated to present the differences between the manual annotations and *pyPPG* fiducial points detection (see figures 4 and 5).

Note that the straight-line appearance of the Bland–Altman results for *on* is due to the times of the reference onsets always being equal to zero, as they were used to define the start time of each pulse wave.

### 3.3. pyPPG and PhysioZoo PPG

The resulting systolic peak detection and fiducial points detection algorithms are packaged into an open-source Python library denoted *pyPPG*. In addition, a user-friendly interface is also implemented in the PhysioZoo Software. In order to ensure that *pyPPG* could process a large dataset without technical issues, we ran it over the full MESA database and reported standard statistics for all biomarkers (see appendix tables A2–A5).

## 4. Discussion

This work is expected to contribute significantly to the scientific field of computerized cardiology, leading to a better understanding of the PPG signal. The *pyPPG* toolbox provides an open-source, scientifically validated and comprehensive resource for PPG time series analysis. To develop *pyPPG* we reviewed and standardized the definition of PPG fiducial points and standard features developed over the past decades. Finally, integrating *pyPPG* in PhysioZoo, we provide a user interface enabling access of this resource to scientists with limited computational skills. Overall, the new resource provides scientists interested in PPG analysis with an exhaustive set of tools supporting their research. The major contribution was the implementation and quantitative validation of a fiducial point detector. The peak detection algorithm in the *pyPPG* toolbox was validated on 19 000 h of continuous PPG data, encompassing more than 91 million reference beats. It performed with an 88.18% *F*_1_-score while processing a 1 h segment in 1.98 s. When evaluated on 3000 manually annotated fiducial points, *pyPPG* had a low MAE and consistently outperformed two other open toolboxes. An additional contribution is the integration of 74 standardized physiological PPG biomarkers within *pyPPG*. The toolbox was made open-source, rendering it the only comprehensive and validated Python library that is publicly accessible. In future work, additional biomarkers such as pulse wave decomposition (Kontaxis *et al* 2020) can be added to further expand the toolbox.

A user-friendly interface is also implemented in the PhysioZoo Software software. This interface enables data visualization, exploration and quantitative analysis of a PPG recording. This novel solution provides researchers and clinicians with a valuable resource for comprehensive and reproducible PPG analysis. Finally, the manual annotations of the 219 recordings, including more than 3000 fiducial points, were made open-access to ensure reproducibility of the results and to enable further investigations and advancements in the field of PPG analysis.

In this study we refined the definitions of fiducial points in an attempt to harmonise the disparate and/or vague definitions found in the literature, and we then used these definitions when annotating reference fiducial points for the assessment of the proposed toolbox. Other toolboxes may be using slightly different definitions of fiducial points and this may indeed be reflected by the results. The performance of the peak detection algorithms was only performed on sleep data from atherosclerosis patients (see appendix tables A2–A5). Therefore the evaluation of *Aboy++* on additional databases would be very beneficial. Another limitation of the work was the focus on the analysis of PPG measured using standard clinical oximeters. Adapting the toolbox to incorporate other PPG sources, such as like earlobe PPG or smartwatches, will be of interest, particularly given the widespread use of the latter. The program had another limitation related to the controversial nature of morphological and physiological characteristics of fiducial points (see appendix figure A2). Hence, creating a standardized toolbox presented a significant challenge. Future research can focus on exploration of the impact of amplitude or time-normalization of waves, or any other normalization technique, on variations in signal characteristics.

The *pyPPG* toolbox enables the analysis of large PPG datasets. This could enable a better understanding of the underlying pathophysiology and etiology of heart diseases. The toolbox may also provide valuable features to train machine learning algorithms towards specific diagnosis and risk-prediction tasks. This can be achieved using the following methodology: for short PPG recordings, features can be engineered across the entire recording and used as input for a classifier. For long-term PPG recordings, given that features are typically derived from short time windows rather than from the entire recording, the features can be engineered over shorter windows. Summary statistics capturing both the central tendency and dispersion of a specific feature can be computed. These summary statistics can then be included as features in the model. As such, the *pyPPG* toolbox is a valuable tool which can be used in many physiological and data-driven PPG-related research.

In conclusion, this work provides a standardized and advanced toolbox for the analysis of PPG. Studying the PPG time-series variability using *pyPPG* can enhance our understanding of the manifestations and etiology of diseases. This toolbox can also be used for biomarker engineering in training data-driven models.

## Data Availability

All data that support the findings of this study are included within the article (and any supplementary information files). The MESA and the PPG-BP databases can be accessed via the following links: – MESA: https://doi.org/10.25822/n7hq-c406
– PPG-BP Database: https://doi.org/10.6084/m9.figshare.5459299.v5

## Code and benchmarking availability

The source code, the annotations of the fiducial points, and the results of benchmarked toolboxes are available at http://physiozoo.com and http://pyppg.readthedocs.io websites. The fiducial point annotations and benchmarking results are accessible at https://doi.org/10.5281/zenodo.10523285.

## Appendix

The appendix includes figure A1 illustrating default pyPPG prefiltering, figure A2 comparing high and low-quality PPG' and PPG'' signals, table A1 showing the inter-annotator differences for fiducial points, and tables A2–A5 providing general statistics for each biomarker.

**Table A1.** Inter-annotator differences for fiducial points.

Fiducial point	*sp*	*on*	*dn*	*u*	*v*	*w*	*a*	*b*	*c*	*d*	*e*	*f*	*p*_1_	*p*_*2*_
No. excluded points^a^	0	0	29	0	0	1	0	0	7	7	0	0	0	2
MAE (SD) ms	3(4)	3(4)	4(5)	1(1)	2(2)	2(3)	2(2)	3(2)	3(3)	2(3)	2(2)	2(3)	1(1)	2(2)

^a^ This number refers to fiducial points that have been excluded due to unclear or ambiguous annotations. The mean and standard deviation of the absolute errors (MAE and SD respectively) are reported for each fiducial in ms.

**Table A2.** Summary statistics for biomarkers of PPG signal from the MESA database for 2054 PPG recordings.

PPG signal										
Biomarker	AVG	MED	SD	Q1	Q3	IQR	SKW	KUR	MAD	Unit
***T***_*pi*_	0.85	0.83	0.15	0.79	0.88	0.10	3.19	29.44	0.09	s
***T***_*pp*_	0.85	0.83	0.14	0.79	0.88	0.09	2.71	24.63	0.08	s
***T***_*sys*_	0.38	0.36	0.07	0.34	0.39	0.05	4.08	38.56	0.04	s
***T***_*dia*_	0.47	0.47	0.13	0.41	0.51	0.10	1.89	20.18	0.08	s
***T***_*sp*_	0.21	0.20	0.05	0.18	0.22	0.04	4.50	46.99	0.03	s
***T***_*dp*_	0.38	0.36	0.07	0.34	0.39	0.05	4.08	38.56	0.04	s
Δ ***T***	0.17	0.16	0.05	0.14	0.19	0.05	2.12	14.02	0.03	s
***T***_*sw*10_	0.16	0.16	0.04	0.14	0.18	0.04	2.96	27.86	0.03	s
***T***_*sw*25_	0.14	0.14	0.04	0.12	0.16	0.03	2.68	26.06	0.02	s
***T***_*sw*33_	0.13	0.13	0.04	0.11	0.15	0.03	2.57	25.51	0.02	s
***T***_*sw*50_	0.11	0.11	0.03	0.10	0.13	0.03	2.32	23.75	0.02	s
***T***_*sw*66_	0.10	0.09	0.03	0.08	0.11	0.03	2.12	22.20	0.02	s
***T***_*sw*75_	0.08	0.08	0.03	0.07	0.10	0.03	2.11	22.22	0.02	s
***T***_*sw*90_	0.06	0.05	0.02	0.04	0.06	0.02	2.45	23.71	0.01	s
***T***_*dw*10_	0.50	0.50	0.13	0.44	0.55	0.11	1.39	16.16	0.08	s
***T***_*dw*25_	0.38	0.37	0.12	0.33	0.42	0.09	2.04	18.59	0.08	s
***T***_*dw*33_	0.33	0.32	0.12	0.27	0.36	0.09	2.30	19.30	0.07	s
***T***_*dw*50_	0.23	0.21	0.10	0.18	0.25	0.07	3.21	24.36	0.06	s
***T***_*dw*66_	0.16	0.14	0.09	0.12	0.17	0.05	4.51	38.24	0.05	s
***T***_*dw*75_	0.13	0.11	0.08	0.09	0.13	0.04	5.18	47.43	0.04	s
***T***_*dw*90_	0.07	0.06	0.07	0.05	0.07	0.02	6.14	63.89	0.03	s
***T***_*pw*10_	0.67	0.66	0.14	0.61	0.72	0.11	1.60	18.13	0.09	s
***T***_*pw*25_	0.52	0.51	0.13	0.47	0.56	0.10	2.05	19.56	0.08	s
***T***_*pw*33_	0.46	0.45	0.12	0.40	0.50	0.10	2.22	19.76	0.08	s
***T***_*pw*50_	0.35	0.33	0.11	0.29	0.38	0.08	2.86	22.45	0.07	s
***T***_*pw*66_	0.25	0.24	0.10	0.21	0.27	0.06	4.00	34.20	0.05	s
***T***_*pw*75_	0.21	0.19	0.09	0.17	0.22	0.04	4.58	42.41	0.05	s
***T***_*pw*90_	0.13	0.12	0.08	0.10	0.14	0.04	5.18	53.15	0.04	s
***A***_*sp*_	0.20	0.18	0.12	0.14	0.22	0.07	3.50	34.29	0.06	nu
${{\boldsymbol{A}}}_{{dn}}$	0.10	0.10	0.10	0.07	0.13	0.06	1.01	34.45	0.05	nu
***A***_*dp*_	0.09	0.08	0.09	0.06	0.11	0.05	2.31	34.98	0.05	nu
***A***_*off*_	0.00	0.00	0.07	−0.01	0.01	0.02	1.03	40.12	0.03	nu
***A***_*pi*_	9.50	9.71	97.74	8.64	10.48	1.83	−0.27	162.91	13.21	nu
***A***_*sys*_	4.37	4.33	23.77	3.76	4.97	1.20	−0.24	148.02	3.49	nu
***A***_*dia*_	5.13	5.21	74.42	4.06	6.18	2.12	−0.18	158.93	10.21	nu

Average (AVG); median (MED); standard deviation (SD); lower and upper quartiles (Q1, Q3); inter-quartile range (IQR); Skewness (SKW, indicating a lack of symmetry in the distribution; Kurtosis (KUR, indicating the pointedness of a peak in the distribution curve); and the average difference between the mean and each data value (MAD).

**Table A3.** Summary statistics for biomarkers of signal ratios from the MESA database for 2054 PPG recordings.

Signal ratios										
Biomarker	AVG	MED	SD	Q1	Q3	IQR	SKW	KUR	MAD	Unit
***IPR***	73.45	73.33	9.87	69.33	77.50	8.16	0.41	12.57	6.53	%
***T***_*sys*_/***T***_*dia*_	91.33	81.22	39.32	71.41	98.41	27.00	393.22	2923.76	24.38	%
***T***_*pw*25_/***T***_*pi*_	62.00	62.75	10.52	57.08	68.00	10.91	−85.54	610.45	7.62	%
***T***_*pw*50_/***T***_*pi*_	40.87	39.97	9.85	35.34	45.27	9.93	78.04	627.29	7.09	%
***T***_*pw*75_/***T***_*pi*_	24.74	23.54	7.83	21.02	26.51	5.49	247.90	1915.65	4.88	%
***T***_*pw*25_/***T***_*sp*_	266.17	260.62	76.87	226.93	294.03	67.10	174.35	1698.59	50.18	%
***T***_*pw*50_/***T***_*sp*_	174.02	165.78	61.20	144.75	189.08	44.33	289.07	2596.04	36.91	%
***T***_*pw*75_/***T***_*sp*_	105.68	98.58	47.91	86.73	111.63	24.90	435.03	4669.79	24.71	%
***T***_*dw*10_/***T***_*sw*10_	336.94	323.76	132.82	271.42	380.80	109.38	250.74	2289.27	82.14	%
***T***_*dw*25_/***T***_*sw*25_	298.33	277.70	148.79	229.72	333.07	103.35	355.27	3273.42	84.17	%
***T***_*dw*33_/***T***_*sw*33_	277.89	252.94	155.82	207.23	308.78	101.55	400.58	3716.32	85.66	%
***T***_*dw*50_/***T***_*sw*50_	230.45	195.56	176.10	159.28	246.41	87.13	519.26	5062.43	86.84	%
***T***_*dw*66_/***T***_*sw*66_	197.33	154.47	212.89	123.35	199.78	76.43	634.82	6779.45	91.84	%
***T***_*dw*75_/***T***_*sw*75_	187.13	137.75	244.73	108.57	182.58	74.01	685.40	7569.23	99.48	%
***T***_*dw*90_/***T***_*sw*90_	185.76	114.54	373.62	90.54	154.42	63.88	800.79	9758.14	132.09	%
***T***_*sp*_/***T***_*pi*_	24.80	24.11	5.44	21.71	26.99	5.28	195.60	1503.32	3.80	%
***A***_*sp*_/*A*_*off*_	−251.50	−225.29	5093.80	−265.99	−190.83	75.16	−79.27	15 171.47	673.97	%
***A***_*dp*_/*A*_*sp*_	50.65	47.32	306.54	37.37	56.42	19.05	−132.42	8856.26	54.04	%
***IPA***	1.35	1.24	19.00	0.95	1.50	0.55	0.21	146.18	2.53	nu
***T***_*sp*_/*A*_*sp*_	2.59	1.89	41.34	1.41	2.63	1.22	5.04	118.11	6.17	nu
***A***_*sp*_/Δ ***T***	1.32	1.13	1.06	0.87	1.48	0.61	4.26	43.19	0.54	nu
***A***_*sp*_/(***T***_*pi*_ − ***T***_*sp*_)	0.32	0.28	0.21	0.23	0.36	0.13	3.78	36.18	0.11	nu

Average (AVG); median (MED); standard deviation (SD); lower and upper quartiles (Q1, Q3); inter-quartile range (IQR); Skewness (SKW, indicating a lack of symmetry in the distribution; Kurtosis (KUR, indicating the pointedness of a peak in the distribution curve); and the average difference between the mean and each data value (MAD).

**Table A4.** Summary statistics for biomarkers of PPG derivative from the MESA database for 2054 PPG recordings.

PPG derivatives										
Biomarker	AVG	MED	SD	Q1	Q3	IQR	SKW	KUR	MAD	Unit
***T***_*u*_	0.11	0.08	0.11	0.06	0.11	0.05	6.21	62.13	0.06	s
***T***_*v*_	0.38	0.33	0.15	0.29	0.42	0.13	3.24	20.96	0.11	s
***T***_*w*_	0.42	0.37	0.16	0.32	0.47	0.14	2.80	15.51	0.11	s
***T***_*a*_	0.06	0.04	0.08	0.03	0.07	0.04	6.05	60.85	0.04	s
***T***_*b*_	0.11	0.09	0.08	0.08	0.12	0.04	5.33	50.57	0.05	s
***T***_*c*_	0.15	0.13	0.09	0.11	0.17	0.06	4.04	32.39	0.05	s
***T***_*d*_	0.22	0.21	0.11	0.16	0.27	0.11	2.02	11.56	0.07	s
***T***_*e*_	0.35	0.34	0.11	0.30	0.39	0.09	1.38	13.26	0.07	s
***T***_*f*_	0.38	0.37	0.11	0.33	0.43	0.10	1.33	11.52	0.08	s
***T***_*b*−*c*_	0.04	0.03	0.02	0.02	0.04	0.02	0.87	1.69	0.01	s
***T***_*b*−*d*_	0.11	0.10	0.07	0.06	0.15	0.09	1.28	5.24	0.05	s
***T***_*p*1_	0.13	0.11	0.08	0.09	0.14	0.05	4.92	44.11	0.05	s
***T***_*p*2_	0.21	0.19	0.10	0.14	0.25	0.11	2.31	13.10	0.07	s
***T***_*p*1−*dp*_	0.25	0.25	0.08	0.22	0.28	0.06	−0.41	19.47	0.05	s
***T***_*p*2−*dp*_	0.17	0.17	0.10	0.11	0.23	0.11	−0.57	7.90	0.07	s

Average (AVG); median (MED); standard deviation (SD); lower and upper quartiles (Q1, Q3); inter-quartile range (IQR); Skewness (SKW, indicating a lack of symmetry in the distribution; Kurtosis (KUR, indicating the pointedness of a peak in the distribution curve); and the average difference between the mean and each data value (MAD).

**Table A5.** Summary statistics for biomarkers of PPG derivative from the MESA database for 2054 PPG recordings.

Derivatives Ratios										
Biomarker	AVG	MED	SD	Q1	Q3	IQR	SKW	KUR	MAD	Unit
***T***_*u*_/***T***_*pi*_	11.18	8.44	11.57	7.32	9.95	2.63	498.54	2700.88	5.23	%
***T***_*v*_/***T***_*pi*_	49.82	41.88	19.96	34.71	63.47	28.76	82.65	−12.92	16.85	%
***T***_*w*_/***T***_*pi*_	54.02	45.93	20.01	38.84	67.60	28.76	80.94	−12.58	16.85	%
***T***_*a*_/***T***_*pi*_	5.80	4.07	7.96	2.59	5.52	2.94	547.31	3988.16	3.88	%
***T***_*b*_/***T***_*pi*_	11.42	10.28	8.04	8.44	11.72	3.28	539.86	4105.76	3.88	%
***T***_*c*_/***T***_*pi*_	15.07	14.05	8.46	11.35	16.66	5.31	469.69	3478.37	4.59	%
***T***_*d*_/***T***_*pi*_	25.59	23.16	12.71	16.50	32.05	15.55	134.11	393.65	9.83	%
***T***_*e*_/***T***_*pi*_	38.89	40.33	13.68	31.87	47.13	15.26	−3.42	169.26	10.24	%
***T***_*f*_/***T***_*pi*_	43.02	44.63	13.63	35.81	51.29	15.48	−2.06	162.95	10.28	%
(***T***_*u*_ − *T*_*a*_)/*T*_*pi*_	5.33	3.53	8.88	2.96	6.07	3.12	550.25	4378.08	3.95	%
(***T***_*v*_ − *T*_*b*_)/*T*_*pi*_	38.33	30.90	19.59	24.38	52.20	27.82	69.31	−26.89	16.40	%
***A***_*u*_/*A*_*sp*_	11.44	11.29	10.85	9.85	12.91	3.06	−342.27	19 223.85	3.01	%
***A***_*v*_/*A*_*u*_	−56.53	−50.35	31.53	−65.01	−40.28	24.74	−581.35	6066.96	17.48	%
***A***_*w*_/*A*_*u*_	8.39	6.60	21.72	−5.48	21.43	26.91	−23.67	268.64	16.59	%
***A***_*b*_/*A*_*a*_	−67.51	−71.60	62.78	−96.23	−41.67	54.56	−366.50	5557.12	39.35	%
***A***_*c*_/*A*_*a*_	8.73	6.21	42.85	−14.34	30.65	44.99	151.81	1769.14	30.31	%
***A***_*d*_/*A*_*a*_	−75.06	−67.10	68.71	−94.36	−48.42	45.95	−515.47	7369.81	36.15	%
***A***_*e*_/*A*_*a*_	76.99	66.61	88.58	46.56	90.78	44.22	916.64	14 471.11	32.28	%
***A***_*f*_/*A*_*a*_	−56.11	−48.07	68.51	−70.78	−25.96	44.82	−576.39	6746.56	33.40	%
***A***_*p*2_/***A***_*p*1_	29.51	127.29	1310.75	24.64	204.19	179.55	−845.24	12 641.48	370.00	%
(***A***_*c*_ − *A*_*b*_)/*A*_*a*_	76.24	69.03	75.93	30.69	107.35	76.65	559.28	7767.55	46.26	%
(***A***_*d*_ − *A*_*b*_)/*A*_*a*_	−7.55	6.35	69.52	−33.05	34.80	67.84	−152.71	442.67	50.05	%
***AGI***	−78.17	−69.51	112.94	−113.36	−28.24	85.11	−706.72	11 560.28	57.14	%
${{\boldsymbol{AGI}}}_{{mod}}$	−1.18	−9.10	66.72	−44.11	42.13	86.23	45.44	152.19	52.43	%
${{\boldsymbol{AGI}}}_{{\inf }}$	−144.50	−130.56	132.13	−172.72	−99.70	73.01	−831.10	13 837.36	54.63	%
***AI***	42.33	38.33	84.63	17.14	57.40	40.26	924.79	13 287.84	36.73	%
***RI***_*p*1_	132.42	111.38	2657.66	83.44	154.65	71.21	325.91	17 081.63	504.55	%
***RI***_*p*2_	132.42	111.38	2657.66	83.44	154.65	71.21	325.91	17 081.63	504.55	%
***SC***	0.00	0.00	0.00	0.00	0.00	0.00	−16.01	271.47	0.00	nu
***IPAD***	1.93	0.43	29.07	−0.01	0.79	0.80	10.28	235.07	3.64	nu

Average (AVG); median (MED); standard deviation (SD); lower and upper quartiles (Q1, Q3); inter-quartile range (IQR); Skewness (SKW, indicating a lack of symmetry in the distribution; Kurtosis (KUR, indicating the pointedness of a peak in the distribution curve); and the average difference between the mean and each data value (MAD).
